# Burden of Difficult-to-Treat Resistant Organisms in Gram-Negative Bloodstream Infections: A Retrospective Observational Study From Eastern India

**DOI:** 10.7759/cureus.111986

**Published:** 2026-07-03

**Authors:** Nirmala Poddar, B. Prince, Shruti Patel, Nishikanta Muduli, A. Susanna, Anwesha Bhuyan, Ipsa Mohapatra, Amresh Pati, Dipti Pattnaik

**Affiliations:** 1 Department of Microbiology, Kalinga Institute of Medical Sciences, Bhubaneswar, IND; 2 Department of Community Medicine, Kalinga Institute of Medical Sciences, Bhubaneswar, IND

**Keywords:** antimicrobial susceptibility pattern, difficult to treat resistance, gram negative bacteria, gram negative blood stream infections, multi-drug resistance

## Abstract

Background

Gram-negative bacteria (GNB) are responsible for about one-third to nearly half of adult bloodstream infections, resulting in higher rates of morbidity and mortality. Difficult-to-treat resistance (DTR), among GNB, is characterised as being intermediate or resistant to all β-lactam classes (including carbapenems) and fluoroquinolones, thus constituting a clinically significant resistant phenotype. This study aims to ascertain the prevalence and antibiotic susceptibility pattern of DTR organisms isolated from Gram-negative bloodstream infections (GNBSIs) at a tertiary-level hospital in eastern India.

Methods

This retrospective observational study was conducted in the Department of Microbiology of a tertiary care hospital in eastern India from June 2024 to May 2025. Blood samples were collected in age-specific blood culture bottles and incubated at 37°C. Positive blood cultures were subcultured to obtain pure isolates. Identification and antimicrobial susceptibility testing (AST) were performed using the VITEK 2 Compact automated system. The results were interpreted according to Clinical Laboratory and Standards Institute (CLSI) M100, 2026, 36th edition. Demographic characteristics, comorbidities, risk factors, and in-hospital mortality rates were collected from hospital records. Univariate and multivariate analyses were used to test the association of the variables with DTR GNBSIs.

Results

Among 202 patients with GNBSIs, 67 (33.17%, 95% CI: 26.7-39.7%) exhibited the characteristics of DTR. The most common DTR pathogens were *Klebsiella pneumoniae,* 27 (40.3%, 95% CI: 28.6-52.0%), followed by *Acinetobacter baumannii,* 19 (28.36%, 95% CI: 17.6-39.2%), and *Escherichia coli,* 10 (14.9%, 95% CI: 6.4-23.5%). The majority of the DTR organisms from GNBSIs were isolated from male patients (41, 61.19%) as compared to female patients (26, 38.81%), with maximum occurrence in the age group >60 years (25, 37.3%). Factors such as arterial line, liver disease, dialysis, prior antibiotic use, and prior carbapenem use exhibited statistically significant associations with DTR GNBSIs in univariate analysis. Prior antibiotic use was identified as the independent predictor of DTR GNBSIs in multivariate analysis. AST revealed maximum resistance of DTR isolates to amikacin (39/63, 61.9%) followed by gentamicin (31/59, 52.54%) and co-trimoxazole (29/59, 49.15%). In terms of susceptibility, maximum susceptibility was marked towards tigecycline (37/44, 84.09%), followed by co-trimoxazole (30/59, 50.85%) and gentamicin (28/59, 47.46%), with (63/63, 100%) of the DTR isolates being intermediate to colistin.

Conclusion

The occurrence of DTR and their non-susceptibility to first-line drugs raises serious concern due to the limited availability of effective treatment options. Therefore, active surveillance, strict infection control practices, and antibiotic stewardship are necessary to tackle this situation in hospitals.

## Introduction

In today’s age, antimicrobial resistance (AMR) has emerged as one of the most serious threats to modern healthcare, with limited safe and effective therapeutic options [1]. In fact, the emergence of difficult-to-treat resistant Gram-negative infections often necessitates the use of reserve antibiotics (e.g., aminoglycosides, polymyxins, and tigecycline) [2,3]. Furthermore, it has been reported that Gram-negative bacteria (GNB) account for 25-45% of bloodstream infections (BSIs). However, the exact percentages may vary by region, age group, and healthcare setting [4,5].

However, the rapid dissemination of resistant strains within healthcare settings has led to an increase in drug-resistant infections, often resulting in prolonged hospital stays, increased healthcare costs, and higher mortality rates. Multi-drug resistance (MDR) is defined as “non-susceptibility to at least one agent in >3 antimicrobial classes”. However, classifying isolates using this definition is entirely based on the organism’s in vitro susceptibility to any antibiotic, irrespective of its therapeutic efficacy, pharmacological properties, and real-life applications in treatment. However, difficult-to-treat resistance (DTR), a novel and more clinically relevant classification of antibiotic-resistant phenotype that has been proposed by Kadri et al. [2], is defined as “intermediate (I) or resistant (R) to all reported agents within the carbapenem, other β-lactam, and fluoroquinolone categories”. In simple terms, these isolates are non-susceptible to all first-line antibiotics, necessitating the use of second-line drugs that exhibit poor pharmacological activity and carry an increased risk of toxicity, with anticipated poor outcomes [6].

Driven by the consequences of DTR infections, we are compelled to consider the application of reserve drugs like colistin, polymyxin-B, tigecycline, and aminoglycosides in therapeutics [7,8]. A recent study by Naveenraj et al. [8] reported an incidence of 37.9% DTR Gram-negative bloodstream infections (GNBSIs), with 82% of the carbapenem-resistant GNBSIs exhibiting a DTR pattern in India. A multicentric surveillance from Peru revealed significant resistance levels in GNBSI, with 69% and 10% of the isolates being MDR and DTR, respectively. The overall in-hospital case-fatality ratio for GNBSIs was 33.3% [9].

In India, various studies and national surveillance have recorded exceedingly elevated rates of carbapenem-resistant BSIs due to Enterobacterales and *Acinetobacter* species. However, a study by Prince et al. [10] reported a prevalence of 49% MDR and 27% DTR *Pseudomonas aeruginosa* in intensive care units (ICUs). Nevertheless, data specifically pertaining to DTR GNBSIs is still scarce [11,12]. The current retrospective observational study was conducted to report the prevalence of DTR GNBSIs and the distribution of organisms at a tertiary care centre in eastern India over a period of one year. This study also evaluated the antibiotic susceptibility profile of DTR isolates to reserve drugs, the association of demographic variables, risk factors, co-morbidities, and outcome with DTR GNBSIs using univariate and multivariate analyses.

## Materials and methods

Study design and study population

This retrospective observational study was carried out after obtaining prior approval from the Institutional Ethical Committee (KIIT/KIMS/IEC/2427/2026), during June 2024-May 2025 in a tertiary-care hospital, in Bhubaneswar, Odisha. During the study period, all inpatients, regardless of age or gender, with at least one positive blood culture were included in the study. Blood cultures that yielded organisms deemed contaminants (e.g., typical skin commensals lacking compatible clinical characteristics) and solitary positive cultures from femoral sites were excluded to reduce the risk of misclassifying contamination as true BSI. To further reduce misinterpretation, only the first isolate was considered in the case of patients exhibiting multiple positive blood cultures from the same organism within seven days of the initial positive culture [13]. DTR was characterised as “intermediate (I) or resistant (R) to all reported agents within the carbapenem, other β-lactam, and fluoroquinolone categories”, utilising the consensus criteria established by Kadri et al. [2,3].

Isolation, identification, and antimicrobial susceptibility testing of organisms

As per routine protocol, blood samples were obtained and sent immediately to the microbiology laboratory. Around 8-10 ml of blood was inoculated into BACT‑ALERT blood culture bottles and incubated at 37°C for up to five days in the BACT‑ALERT 3D (bioMérieux, Marcy‑l'Étoile, France) automated system. Following incubation, positive blood cultures were sub-cultured on blood and MacConkey agar (Himedia Pvt. Ltd, Mumbai, India) to obtain pure isolates. Initial identification was done using Gram staining and routine conventional techniques. Further species‑level identification of Gram-negative organisms was done using the VITEK 2 Compact automated system (bioMérieux, Marcy‑l'Étoile, France) with Gram-negative identification cards, following the manufacturer’s instructions. Antimicrobial susceptibility testing (AST) was performed using the VITEK 2 Compact automated system, and the results were interpreted according to Clinical and Laboratory Standards Institute (CLSI) guidelines M100, 36th edition, 2026 [14]. The antibiotic panel for DTR characterisation included imipenem, meropenem, ceftazidime, cefepime, piperacillin/tazobactam, and ciprofloxacin. Isolates lacking susceptibility testing results in any of these drugs were not designated as DTR [2,3]. Minimum inhibitory concentration (MIC) of colistin was further confirmed by broth microdilution (BMD) method using colistin HiMIC™ plate kit (Himedia Pvt. Ltd, Mumbai, India) as per manufacturer’s instructions. For quality control purposes, *Stenotrophomonas maltophilia* ATCC 17666 (for identification), *Escherichia coli* ATCC 25922, *E. coli* ATCC 35218, and *Klebsiella pneumoniae* ATCC 700603 (for susceptibility testing) were evaluated in parallel with clinical isolates on a weekly basis, according to CLSI recommendations [14].

Data collection and statistical analysis

Data regarding the occurrence and AST pattern of organisms isolated from GNBSIs were obtained from the laboratory information system (LIS) of the hospital. For each patient, the following data were extracted: age, gender, comorbidities including diabetes, hypertension, malignancy, renal disease, cardiac disease, liver disease, prior cerebrovascular accident, risk factors such as ICU exposure, mechanical ventilation, urinary catheter, arterial line, recent surgery, haemodialysis, prior antibiotic use, prior carbapenem use, and duration of hospital stay before BSI. The outcome variable encompassed in-hospital mortality. Normality of the continuous variables was evaluated using the Shapiro-Wilk test. Median and interquartile range were used to summarise skewed data, whereas mean ± SD was reported for normally distributed data. Categorical variables were represented as frequencies and percentages. Chi-square test or Fisher's exact test was used for categorical variables, and the Mann-Whitney U test or independent-samples t-test was used for continuous variables as tests of association. Variables significantly correlated with DTR GNBSIs and other important drivers of infection in univariate analysis were incorporated into a binary logistic regression model to ascertain independent risk factors. Variables with p-value <0.05, such as liver disease, arterial line, dialysis, prior antibiotic use, and prior carbapenem use, along with clinically relevant factor such as age were included in a multivariate logistic regression model. DTR status (DTR=1, Non-DTR=0) was used as the dependent variable. Results were reported as adjusted odds ratios (aORs) with 95% confidence intervals. A p-value <0.05 was considered statistically significant. The multivariable model was restricted to six predictors, yielding approximately 11 events per variable. R software Version 4.4.3 (R Foundation for Statistical Computing, Vienna, Austria) [15] was used to generate figures. Statistical analysis was performed using Jamovi software [16].

## Results

**Figure 1:**
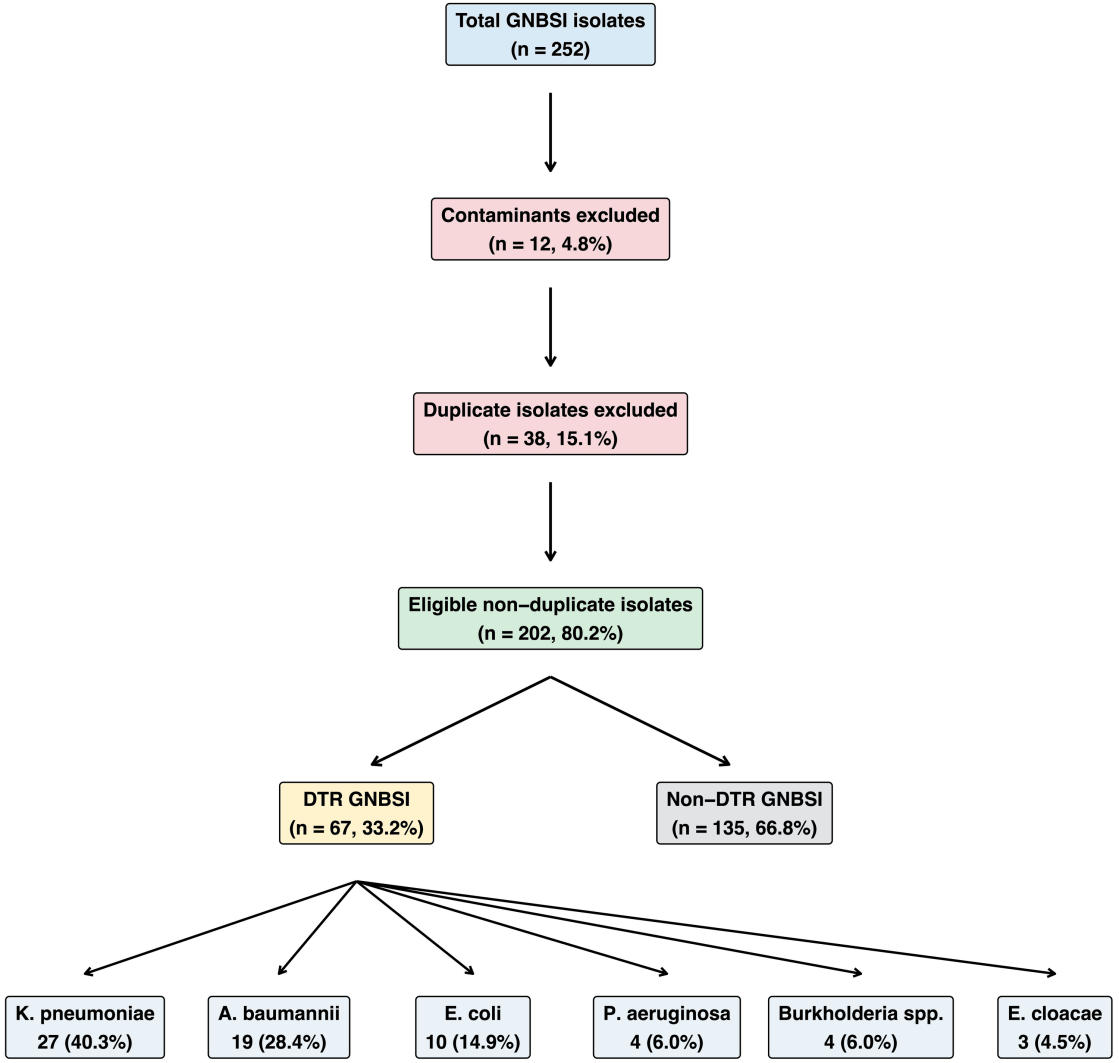
Distribution of Gram-negative organisms among DTR GNBSI cases The data has been represented as (n, %) for all the variables. GNBSI: Gram-negative bloodstream infection; DTR: difficult-to-treat resistant.

During the study period, a total of 252 GNBSI isolates were identified. Of these, 12 (4.8%) contaminants and 38 (15.1%) duplicate isolates, which did not meet the inclusion criteria, were excluded from the study. Finally, 202 (80.2%) non-duplicate isolates were further considered for data interpretation and analysis. Among them, 67 (33.17%, 95% CI: 26.7-39.7%) DTR GNB and 135 (66.83%, 95% CI: 60.3-73.3%) non-DTR GNB were isolated. The prevalence of DTR isolates in GNBSIs was 33.17% (67/202). Within the DTR GNB isolates, *K. pneumoniae* was the most common organism isolated, accounting for 27 (40.3%, 95% CI: 28.6-52.0%), followed by *Acinetobacter baumannii,* 19 (28.36%, 95% CI: 17.6-39.2%), and *E. coli, *10 (14.9%, 95% CI: 6.4-23.5%). Fewer *P. aeruginosa* and other non-fermenters made up the rest, as shown in Figure 1.

Table 1 compares the distribution of organisms between DTR and non-DTR GNBSI groups. Non-DTR organisms comprised 135 (66.83%, 95% CI: 60.3-73.3%) of the total eligible non-duplicate isolates from GNBSIs. The most prevalent organisms in DTR GNBSIs and non-DTR GNBSIs were *K. pneumoniae,* 27 (40.3%, 95% CI: 28.6-52.0%), and *E. coli,* 55 (40.7%, 95% CI: 32.45-49.03%), respectively.

**Table 1:** Distribution of organisms in DTR and non-DTR GNBSIs The occurrence of the organisms in both DTR and non-DTR groups has been represented as (n, %). DTR: difficult-to-treat resistant; GNBSIs: Gram-negative bloodstream infections.

Organism	DTR GNBSIs (n = 67)	%	Non-DTR GNBSIs (n = 135)	%
*K*lebsiella* pneumoniae*	27	40.3	36	26.7
Acinetobacter baumannii	19	28.4	14	10.4
Escherichia coli	10	14.9	55	40.7
Pseudomonas aeruginosa	4	6.0	12	8.9
*Burkholderia* spp.	4	6.0	11	8.1
*Enterobacter cloacae* complex	3	4.5	7	5.2

**Figure 2:**
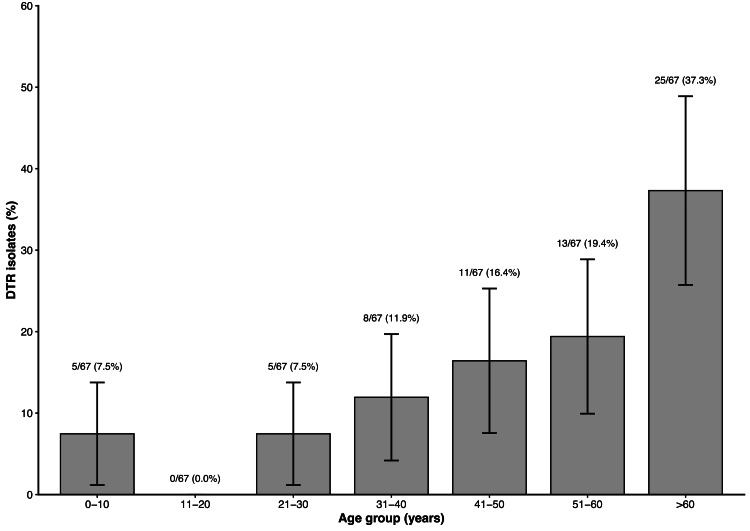
Occurrence of DTR GNB (in percentages) across different age groups The frequency of organisms falling under each age category has been represented as (n, %), along with their 95% confidence intervals represented as error bars. DTR: difficult-to-treat resistant.

Gender-wise distribution revealed that male patients were predominant in both DTR and non-DTR groups. Among DTR GNBSIs, the majority of the isolates were from male patients (61.19%, 41/67) as compared to female patients (38.81%, 26/67). The age was not normally distributed either in the DTR group (Shapiro-Wilk W = 0.934, p = 0.002) or in the non-DTR group (Shapiro-Wilk W = 0.925, p < 0.001). The median age of patients with DTR GNBSIs was 54 years (IQR: 40-66 years). The maximum occurrence (25, 37.3%) was observed in the geriatric cohort (i.e., >60 years), followed by the 51-60 years (13, 19.4%) and the 41-50 years (11, 16.4%) age groups (Figure 2).

Table 2 compares demographic data, co-morbidities, risk factors, and outcomes between patients with DTR GNBSIs and non-DTR GNBSIs. Univariate analysis revealed that patients with liver disease (p = 0.043), arterial line (p = 0.019), dialysis (p = 0.017), prior antibiotic use (p < 0.001), and prior carbapenem use (p = 0.001) were significantly associated with DTR GNBSIs. Although the mortality rate was higher in the case of DTR GNBSIs (33, 49.25%) than in non-DTR GNBSIs (57, 42.22%), the difference was not statistically significant (p = 0.344, OR: 1.33, 95% CI: 0.738-2.39). 

**Table 2:** Association of demographic characteristics, risk factors, co-morbidities, and outcome with DTR and non-DTR GNBSIs ^a^Mann-Whitney U test. ^b^Chi-square test. ^c^Fisher’s exact test. ^d^Independent-sample t-test. Findings for each category for both DTR and non-DTR have been represented as n and column percentages except for “age group” and “duration of hospital stay before BSI”, which have been represented as (median - IOR) and (mean ± SD), respectively. A p-value <0.05 was considered as statistically significant; the statistically significant p-values (i.e. p < 0.05) are represented in bold. DTR: difficult-to-treat resistant; GNBSIs: Gram-negative bloodstream infections; df: degree of freedom; IQR: interquartile range; T2DM: type II diabetes mellitus; HTN: hypertension; CVA: cerebrovascular accident; ICU: intensive care unit; BSI: bloodstream infection; SD: standard deviation.

Demographic characteristics	DTR GNBSIs (n = 67)	Non-DTR GNBSIs (n = 135)	Test statistics	df	p-Value	Odds ratio (95% CI)
Age (median - IQR)	54 (40-66)	52 (31-63)	4004	-	0.185^a^	-
Gender						
Male	41 (61.19%)	81 (60%)	0.0267	1	0.87^b^	0.951 (0.522-1.73)
Female	26 (38.81%)	54 (40%)				
Co-morbidities						
T2DM	13 (19.4%)	31 (22.96%)	0.333	1	0.564^b^	0.808 (0.391-1.67)
HTN	18 (26.87%)	30 (22.22%)	0.533	1	0.465^b^	1.29 (0.654-2.53)
Renal disease	22 (32.84%)	30 (22.22%)	2.64	1	0.104^b^	1.71 (0.892-3.28)
Cardiac disease	5 (7.46%)	9 (6.67%)	0.044	1	0.834^b^	1.13 (0.363-3.51)
Liver disease	25 (37.31%)	32 (23.7%)	4.09	1	0.043^b^	1.92 (1.02-3.61)
Malignancy	8 (11.94%)	14 (10.37%)	0.114	1	0.736^b^	1.17 (0.466-2.95)
CVA	7 (10.45%)	20 (14.81%)	0.737	1	0.39^b^	0.671 (0.269-1.68)
Risk factors						
ICU exposure	49 (73.13%)	97 (71.85%)	0.0368	1	0.848^b^	1.07 (0.552-2.06)
Mechanical ventilation	7 (10.45%)	11 (8.15%)	0.292	1	0.589^b^	1.32 (0.486-3.56)
Arterial line	10 (14.93%)	7 (5.19%)	5.51	1	0.019^b^	3.21 (1.16-8.85)
Foley's catheter	10 (14.93%)	34 (25.19%)	2.77	1	0.096^b^	0.521 (0.240-1.13)
Central vein catheter	29 (43.28%)	54 (40%)	0.199	1	0.655^b^	1.14 (0.632-2.07)
Surgery	2 (2.99%)	9 (6.67%)	-	-	0.344^c^	0.431 (0.09-2.05)
Dialysis	7 (10.45%)	3 (2.22%)	-	-	0.017^c^	5.13 (1.28-20.5)
Prior antibiotic use	60 (89.55%)	90 (66.67%)	12.3	1	<0.001^b^	0.233 (0.09-0.55)
Prior carbapenem use	40 (59.7%)	48 (35.56%)	10.6	1	0.001^b^	0.372 (0.20-0.68)
Duration of hospitalisation before BSI (mean ± SD)	11.1 ± 3.55	10.7 ± 3.28	-0.914	200	0.362^d^	Cohen’s d: -0.137 (-0.43 to 0.157)
Septic shock	23 (34.33%)	45 (33.33%)	0.0199	1	0.89^b^	1.05 (0.563-1.94)
Outcome						
Mortality	33 (49.25%)	57 (42.22%)	0.896	1	0.344^b^	1.33 (0.738-2.39)

Multivariate analysis was performed, including significant factors and other important drivers of DTR GNBSIs. In multivariate analysis, prior antibiotic use (p = 0.029, aOR: 0.334, 95% CI: 0.125-0.897) was found to be the independent predictor of DTR GNBSIs. However, variables such as liver disease (p = 0.179, aOR: 1.649, 95% CI: 0.796-3.418), arterial line (p = 0.323, aOR: 1.764, 95% CI: 0.573-5.429), dialysis (p = 0.223, aOR: 2.618, 95% CI: 0.557-12.293), and prior carbapenem use (p = 0.165, aOR: 0.600, 95% CI: 0.292-1.234) were no longer statistically significant after multivariate logistic regression (Table 3).

**Table 3:** Association of different factors with DTR GNBSIs in univariate and multivariate analyses Odds ratios with 95% confidence intervals for univariate and multivariate analyses have been reported for all the variables. A p-value <0.05 was considered as statistically significant; the statistically significant p-value (i.e. p < 0.05) is represented in bold. CI: confidence interval; DTR: difficult-to-treat resistant; GNBSIs: Gram-negative bloodstream infections.

Factor	Univariate analysis	Odds ratio (95% CI)	Multivariate analysis	Adjusted odds ratio (95% CI)
Age	0.185	-	0.280	1.008 (0.993-1.024)
Liver disease	0.043	1.92 (1.02-3.61)	0.179	1.649 (0.796-3.418)
Arterial line	0.019	3.21 (1.16-8.85)	0.323	1.764 (0.573-5.429)
Dialysis	0.017	5.13 (1.28-20.5)	0.223	2.618 (0.557-12.293)
Prior antibiotic use	<0.001	0.233 (0.09-0.55)	0.029	0.334 (0.125-0.897)
Prior carbapenem use	0.001	0.372 (0.20-0.68)	0.165	0.600 (0.292-1.234)

**Figure 3:**
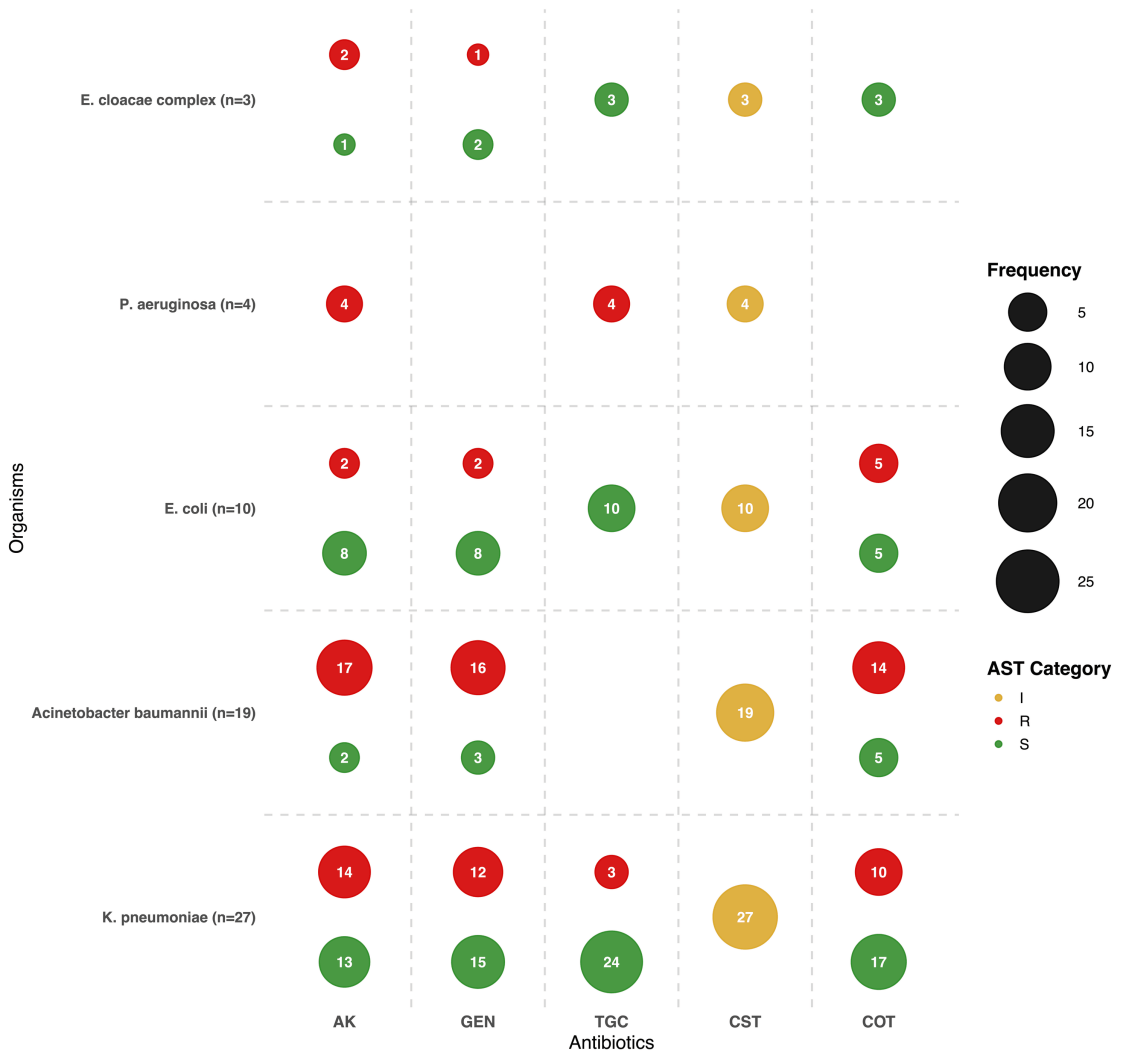
Antibiotic susceptibility pattern of DTR isolates Bubble size represents the frequency, and colours denote susceptibility categories (susceptible, intermediate, and resistant). Blank cells: CLSI interpretive criteria not available. Antimicrobial susceptibility testing was not recommended for *Burkholderia *spp. as per CLSI, M100, 2026. AK: amikacin; GEN: gentamicin; TGC: tigecycline; CST: colistin; COT: co-trimoxazole; I: intermediate; R: resistant; S: susceptible; DTR: difficult-to-treat resistant; CLSI: Clinical Laboratory and Standards Institute.

In the current study, the antibiotic susceptibility profile of DTR isolates showed extensive co-resistance to aminoglycosides and other reserve agents, with relative preservation of activity for colistin. DTR isolates exhibited maximum resistance to amikacin (39/63, 61.9%), followed by gentamicin (31/59, 52.54%) and co-trimoxazole (29/59, 49.15%). In terms of susceptibility, maximum susceptibility was marked towards tigecycline (37/44, 84.09%), followed by co-trimoxazole (30/59, 50.85%) and gentamicin (28/59, 47.46%), with (63/63, 100%) of the DTR isolates being intermediate to colistin. *Klebsiella pneumoniae* (n = 27) exhibited the highest resistance to amikacin, 14 (51.85%), followed by gentamicin, 12 (44.44%), whereas 24 (88.89%) and 27 (100%) were susceptible and intermediate to tigecycline and colistin, respectively. In case of *A. baumannii* (n = 19), 17 (89.47%) and 16 (84.21%) were non-susceptible to amikacin and gentamicin, with all the isolates being intermediate to colistin. However, 8 (80%) of *E. coli* (n = 10) isolates were susceptible to both amikacin and gentamicin (Figure 3).

An additional table has been provided in the Appendix reporting the susceptibility rates of DTR organisms interpreted as per CLSI 2026 guidelines [14].

## Discussion

In this study, the prevalence of DTR GNBSIs was 33.17%, which aligns with the findings of Naveenraj et al. [8], who reported a slightly higher incidence of 37.9% DTR GNBSIs. Another study by Kumar et al. [17] has reported a 20% prevalence of DTR organisms among hospitalised patients. Such a high prevalence demands urgent attention in healthcare settings. However, Kadri et al. (2018) [2] reported a prevalence of 1% DTR GNBSIs among 92 US hospitals during 2009-2013. These variations may reflect differences in study location, population, and time period. In our study, *K. pneumoniae* was the most frequent DTR GNBSI pathogen (40.3%), followed by *A. baumannii* (28.36%) and *E. coli* (14.9%), corresponding to the predominance of *Klebsiella* and *Acinetobacter* among DTR isolates in a study by Naveenraj et al. [8]. However, *A. baumannii* has been reported as a predominant DTR organism in a recent multicentric study in the United States, as well as in a retrospective observational Indian study, underlining its importance in being listed among critical groups in the WHO bacterial priority pathogens list 2024 [18-20].

Gender-wise distribution revealed a higher incidence of DTR GNBSIs in male patients (61.19%) than in female patients (38.81%). Comparable findings of DTR bacteraemia have been reported in US and Indian cohorts [2,8]. The highest occurrence of DTR GNBSIs among the geriatric cohort in this study aligns with the findings of Kadri et al. [2], suggesting a potential risk to patients aged >60 years. Univariate analysis highlighted a significant association of patients with liver disease, arterial line, dialysis, prior antibiotic use, and prior carbapenem use with DTR GNBSIs. After multivariate analysis, prior antibiotic use was identified as the independent predictor of DTR GNBSIs, whereas some studies have reported healthcare-associated infections, ICU stay, and mechanical ventilation to be the independent predictors of DTR GNBSIs [8,21]. These discrepancies may be attributed to differences in patient population, severity of illness, antimicrobial prescribing practices, and geographic locations. Moreover, the elevated prevalence of DTR is probably due to the long-term use of carbapenems and broad-spectrum β-lactams, high background rates of MDR and expanded-spectrum cephalosporin-resistant (ESCR) phenotypes, and strong selection pressures in ICUs and high-dependency units.

Research conducted in India and Peru reports that resistance to third-generation cephalosporins and carbapenems is highly prevalent among GNB bloodstream isolates, with MDR patterns identified in up to 69% of these isolates [3,8,20]. DTR GNBSIs present significant therapeutic difficulties. In this study, 61.9% and 52.54% of the DTR isolates were resistant to amikacin and gentamicin, respectively. Naveenraj et al. [8] in their study reported that 81.8% of DTR isolates exhibited resistance to amikacin, 12.1% to tigecycline, and 4.8% to colistin. Consequently, the DTR infections pose a significant therapeutic challenge owing to the limited availability of effective antimicrobial agents [18].

DTR is better than the old MDR/extensive drug resistance (XDR) classifications in many ways. It concentrates on high-efficacy, low-toxicity first-line agents, rather than tallying resistance categories regardless of clinical utility. It is more straightforward to implement at the bedside than the comprehensive MDR/XDR/ pan drug resistance (PDR) framework [6,22]. Moreover, the overall in-hospital mortality rate for GNBSI was higher, which aligns with various Indian and global studies [3,8,18]. The elevated mortality associated with DTR GNBSIs highlights the need for enhanced access to novel agents. Some new combinations of β-lactam/β-lactamase inhibitors, as well as other drugs like ceftazidime-avibactam, ceftolozane-tazobactam, imipenem-relebactam, meropenem-vaborbactam, cefiderocol, plazomicin, and eravacycline, have been reported to exhibit enhanced in vitro activity against certain types of DTR Enterobacterales and non-fermenters. However, their effectiveness depends on the type of resistance and species [23].

Nonetheless, this study has several limitations. Due to the single-centre design, relatively small sample size, and retrospective nature of the study, the exact epidemiology of GNBSI could not be determined. The lack of comprehensive data regarding the genetic mechanism of resistance genes, incomplete clinical variable definition, the appropriateness of antibiotic treatment, time to active therapy, source control, and long-term outcomes beyond discharge hinders a more detailed analysis. Although the multivariable model was restricted to six predictors, yielding approximately 11 events per variable, the relatively small number of DTR-GNBSI cases may still have limited the precision of the adjusted estimates. Consequently, the results of the multivariable analysis should be interpreted cautiously and validated in larger multicentric studies. The strength of the study is that it reports for the first time the evidence from a tertiary care centre in eastern India. The study helps to identify the prevalence and associated risk factors of DTR. Furthermore, the molecular analysis of the isolates was not performed, which further limits the analysis. However, further multicentric studies with larger sample sizes are required for a better understanding of resistance profiles and the association of potential risk factors and co-morbidities with DTR infections.

## Conclusions

In this study, GNBSIs exhibited characteristics of DTR, predominantly driven by *K. pneumoniae*, *A. baumannii*, and *E. coli*, with their non-susceptibility to first-line drugs. Factors such as arterial line, liver disease, dialysis, prior antibiotic use, and prior carbapenem use exhibited a statistically significant association with DTR GNBSIs in univariate analysis. However, prior antibiotic use was identified as the independent predictor of DTR GNBSIs. The emergence of DTR remains a significant threat to hospitalised patients due to limited therapeutic options. This calls for judicious use of second-line drugs, rapid diagnosis, exploration of non-antibiotic approaches, immediate improvements in surveillance, infection prevention and control, and antimicrobial stewardship, as well as the smart use of new anti-GNB agents when they are available. Future multicentric studies that include molecular characterisation and treatment-outcome correlations are necessary to enhance management strategies for DTR GNBSIs in India and other high-burden areas.

## Appendices

**Table 4:** Susceptibility pattern of DTR organisms All isolates of *P. aeruginosa* were non-susceptible to amikacin and tigecycline and intermediate to colistin. Antibiotic susceptibility rates have been reported as the number of organisms and their percentages susceptible to different antibiotics, interpreted as per CLSI 2026 guidelines. Blank cells: Unavailability of interpretive criteria for specific organism-antibiotic combination in CLSI 2026. DTR: difficult-to-treat resistance; CLSI: Clinical Laboratory and Standards Institute.

Antibiotic	*Klebsiella pneumoniae* (n = 27)	*Acinetobacter baumannii* (n = 19)	*Escherichia coli* (n = 10)	*Enterobacter cloacae* complex (n = 3)
Amikacin	13 (48.15%)	2 (10.53%)	8 (80%)	1 (33.33%)
Gentamicin	15 (55.56%)	3 (15.79%)	8 (80%)	2 (66.67%)
Tigecycline	24 (88.89%)	-	10 (100%)	3 (100%)
Cotrimoxazole	17 (62.96%)	5 (26.32%)	5 (50%)	3 (100%)
